# Supplementary material for: Caspase-2 mRNA levels are not elevated in mild cognitive impairment, Alzheimer’s disease, Huntington’s disease, or Lewy Body dementia
Source: PLoS One. 2022 Sep 21;17(9):e0274784. doi: 10.1371/journal.pone.0274784 (PMC9491574; doi:10.1371/journal.pone.0274784)
Supplement: S1 Table — (DOCX) [file pone.0274784.s001.docx]

**S1 Table**. IDT PrimeTime qPCR probe assays used in this study

| **Gene (Ref Seq)** | **IDT Assay** | **Forward primer** | **Reverse primer** | **Probe sequence^1^** | **Dye^2^** |
| --- | --- | --- | --- | --- | --- |
| CASP2-L (NM_032982) | Hs.PT.56a.4001907 | ATCCTGCGTGGTTCTTTCC | CCCAAGCCTACAGAACAAAC | TGCCGTGGAGATGAGACTGATCGT | FAM |
| CASP2-S (NM_001224) | Hs.PT.56a.18690857 | CACGATCAGTCTCATAGAGCA | CCAAGCCTACAGAACAAACCAA | ATCCAATAGCACCTCCACGGCAG | FAM |
| SNAP25 (NM_003081) | Hs.PT.58.20396111 | CACTTAACCACTTCCCAGCAT | GCAATGAGATCGATACACAGAATC | CCAGAATTGATGAGGCCAACCAACG | FAM |
| TUBB3 (NM_001197181) | Hs.PT.58.20385221 | CCTCCGTGTAGTGACCCTT | GGCCTTTGGACATCTCTTCAG | CGGCCCCACTCTGACCAAAGAT | FAM |
| PPIA (NM_021130) | Hs.PT.58v.38887593 | CAAGACTGAGATGCACAAGTG | GTGGCGGATTTGATCATTTGG | AATTCACGCAGAAGGAACCAGACAGT | HEX |
| RPL13 (NM_000977) | Hs.PT.58.26748094.g | AACAGCCTGCAAGTCATCTAC | TGGAAATAGGTGATGCTGCTAC | AGGTGTCATCGTGTATCAAGTGTGGC | HEX |

1) All qPCR probe assays include mid-sequence ZEN and 3’ Iowa Black FQ quencher dyes which have been omitted for clarity
2) FAM: Fluorescein (Ex 495nm/Em 520nm), HEX: Hexachlorofluorescein (Ex 538nm/Em 555nm)
