# Supplementary material for: Caspase-2 mRNA levels are not elevated in mild cognitive impairment, Alzheimer’s disease, Huntington’s disease, or Lewy Body dementia
Source: PLoS One. 2022 Sep 21;17(9):e0274784. doi: 10.1371/journal.pone.0274784 (PMC9491574; doi:10.1371/journal.pone.0274784)
Supplement: S2 Table — (DOCX) [file pone.0274784.s002.docx]

**S2 Table**. Revised patient demographics of samples with RIN >= 3.9

|  | **AD** | **MCI** | **AD/MCI controls** | **P-value^1^** |  | **LBD** | **LBD controls** | **P-Value^2^** |  | **HD** | **HD controls** | **P-value^2^** |
| --- | --- | --- | --- | --- | --- | --- | --- | --- | --- | --- | --- | --- |
| **Sample Size (N)** | 9 | 9 | 20 |  |  | 9 | 5 |  |  | 11 | 5 |  |
|  |  |  |  |  |  |  |  |  |  |  |  |  |
| **Age** |  |  |  |  |  |  |  |  |  |  |  |  |
| **Mean** | 90.9 | 86.6 | 85.9 | 0.17 |  | 77.1 | 84.4 | 0.2 |  | 63.3 | 72.2 | 0.09 |
| **Range** | 85-98 | 75-94 | 91-94 |  |  | 57-96 | 80-92 |  |  | 50-73 | 57-79 |  |
|  |  |  |  |  |  |  |  |  |  |  |  |  |
| **Sex** |  |  |  |  |  |  |  |  |  |  |  |  |
| **Male** | 2 | 5 | 7 | 0.35 |  | 7 | 3 | 0.6 |  | 3 | 4 | 0.06 |
| **Female** | 7 | 4 | 13 |  |  | 2 | 2 |  |  | 8 | 1 |  |
|  |  |  |  |  |  |  |  |  |  |  |  |  |
| **PMI** |  |  |  |  |  |  |  |  |  |  |  |  |
| **Mean** | 11.4 | 12.2 | 11.0 | 0.92 |  | 6.0 | 5.9 | 1.0 |  | 12.6 | 13.5 | 0.6 |
| **Range** | 2-24 | 2-28 | 2-23 |  |  | 2-16.3 | 1.9-11 |  |  | 6-20.3 | 11-17.8 |  |

1. One-way ANOVA
2. Two-tailed T test
