# Supplementary material for: Caspase-2 mRNA levels are not elevated in mild cognitive impairment, Alzheimer’s disease, Huntington’s disease, or Lewy Body dementia
Source: PLoS One. 2022 Sep 21;17(9):e0274784. doi: 10.1371/journal.pone.0274784 (PMC9491574; doi:10.1371/journal.pone.0274784)
Supplement: Attachment — Submitted filename: Response To Reviewers.docx [file pone.0274784.s004.docx]

**Manuscript PONE-D-22-13921**

**Response To Reviewers**

Dear Dr. Bhat,

Thank you for the opportunity to submit a revised version of the manuscript now titled “Caspase-2 mRNA Levels Are Not Elevated in Mild Cognitive Impairment, Alzheimer’s Disease, Huntington’s Disease, or Lewy Body Dementia” to PLOS ONE. We would like to thank the Editor and Reviewers for taking the time to review our manuscript and appreciate the insightful comments that improved this manuscript. Please see our responses to the reviewers below.

**Reviewer 1**

1. In the methods line 169 it stats P-values > 0.05 are statistically different, this should be <.

Thank you for catching this mistake. We have corrected the error in the resubmitted manuscript.

2. In the table (or generation of another table) it would be good to show the new n for each group after the cut off for RIN 3.9. this should then also include the sex of the patients, could also give the new age variation too.

We agree and have submitted a supplementary table to include this information.

**Reviewer 2**

1: The title of this paper is too broad and the conclusion drawn from this single study is exaggerated. Please consider narrowing down your findings and phrase appropriately.

We agree with Reviewer 2 that the initial version of the paper was too broad. We have amended the title to reflect the changes suggested. We have also narrowed the conclusion to include only the diseases and samples that we tested here (Title Page and Conclusion Section (Page 18).

2: The CASP2 mRNA assessment needs an alternative approach that is different from traditional qRT-PCR. Although the authors claimed a good rationale and strategy to exclude highly degraded RNA samples (RIM <3.9), however, this also biased excluded many samples from the total population. Considering the low quality of this RNA (RINs: 1.2-6.1), I would highly recommend a different approach to measure the CASP2 transcript level in all samples, such as nCounter digital imaging of RNA molecules developed by NanoString. It's even possible to simultaneously detect the protein and mRNA of CASP2 in the same samples.

We would like to thank Reviewer 2 for bringing nCounter to our attention. We explored the possibility of using nCounter and were told that while it is very good at measuring RNA in multiple stages of degradation; the system is for multiplexing many RNA targets, not just 1 and a housekeeping gene. We were told the current extraction would not be suitable for nCounter. Unfortunately, we have exhausted many of the samples that were used for this, and previous, publications and can not repeat the experiment using a different method. In the future, we will explore the possibility of using new, more advanced methods.

We initially hypothesized that there would be increases in caspase-2 mRNA levels due to previously published literature that used rtPCR. We believe it is important to disprove this hypothesis using the same techniques as papers used in the past.

Q3: The authors discussed the potential role of miRNAs in increasing Caspase-2 protein in respect of the unchanged mRNA level. Can you give a few examples to address this point further? Are you available to measure some of these critical miRNAs in your samples?

This is an excellent point brought up by Reviewer number 2. We have expanded on the potential roles of miRNA in regulating caspase-2 protein levels (Page 16). We would like to research this hypothesis in the future to discover the precise roles of miRNAs in neurodegeneration. Unfortunately, that will have to be on a new set of samples when they can be obtained. Additionally, we feel this is beyond the scope of this paper, which is to report the discrepancies of our findings to published works and to disprove hypotheses that were discussion points in our previous papers.

Q4: The authors discussed that protein trafficking from the nucleus, mitochondria, and ER might have contributed to the increased cytosol accumulation of Caspase-2 in these diseases. Are you able to perform subcellular protein fractionation for these samples to validate this?

We agree with Reviewer 2 that this would be an excellent experiment that will add to the understanding of caspase-2 biology. Unfortunately, we do not have enough of the brain samples used for this study to do these experiments.
